# Perchloride of Iron in Post-Partum Hæmorrhage

**Published:** 1874-04-15

**Authors:** 


### PERCHLORIDE OF IRON IN POST-PARTUM HÆMORRHAGE.

*Editorial in The Obstetrical Journal of Great Britain and Ireland.*

DURING the last twelve months, much space in this *Journal* has been devoted to the consideration and discussion of the treatment of post-partum hæmorrhage, and more particularly to the method so ably advocated and defended by Dr. Robert Barnes. Post-partum hæmorrhage, perhaps more than any other accident, claims the attention and arouses the interest of the medical practitioner. When it occurs, his nerve, energies, and resources, are strained to their utmost limits, and, consequently, an indellible impression is produced. At all medical meetings, when this subject is brought forward, animated discussions invariably follow. Every one wishes to ventilate his experience, and to learn something new and potent upon which he may rely when next he has the misfortune to battle with a case. This prevalent feeling was probably the cause why the profession seized so greedily upon the perchloride of iron remedy. A weapon, with which death in its most appalling form could be conquered, was what every obstetrician and general practitioner wanted. Many are wielding it now; and whether it be trusty or no, will speedily come to light. So far, the opinions of those who have tried it are almost unanimously favorable. Those who doubt its efficacy, or think it dangerous, are represented in this country by Dr. Snow Beck, and in France by Dr. Joulin. It is well known that a solution of perchloride of iron injected into a nevus has, by producing clots in the blood-vessels, caused death in a few minutes. But this is not the form of accident which the objectors to its use most dread. Blood-poisoning, they assert, is the real danger—septicemia, with its long train of fatal symptoms. Whether there be any truth in their strongly expressed opinions, time alone can decide. Whatever, however, may be said against iron injections in post-partum hæmorrhage, we think it must be conceded that many lives have been, and are being, saved by them. The questions as to whether any other better, or equally effective, method of treating this form of hæmorrhage is discoverable, and whether iron is the safest and best form of styptic, are still open; but that Dr. Barnes' plan has been of the greatest service in many desperate cases there can be no reasonable doubt. He therefore deserves the thanks of the profession for urging the use of a remedy which, if not the best, is at least the only certainly effectual one at present known, to which we can turn when all other expedients have failed. The chief source of danger in using the perchloride of iron lies in the power which it possesses of forming the blood into dense clots. The decomposition of these, whether they be in the sinuses or cavity of the uterus, is the pathogenetic consequence most to be feared. To prevent the deep penetration of the iron into the sinuses, Dr. Wynn Williams swabs instead of injecting. To avoid danger in the second case, it is essential, as pointed out by Dr. Barnes, that, both before and after the iron injection, the cavity of the uterus should be completely emptied. To enable the profession to form a just estimate of the value of this method of treating post-partum hæmorrhage, and to obtain perfect confidence in it, the publication of all cases, whether favorable or the reverse, is very desirable. We hope Dr. Snow Beck will publish his cases, and Dr. Barnes, in ever so brief a form, the whole of his. We also hope that the flourishing accounts of the successful cases which have appeared in our pages, will not induce any one to use the perchloride of iron injection, or swabbing, without having first tried the ordinary means; and that no one will be deterred, when these have failed, from promptly employing it, and thus giving his patient a last chance, on account of anything against its use, which we, in our impartial position, have thought it our duty to publish.

